# Surgical vs. Medical Management of Infective Endocarditis Following TAVR: A Systematic Review and Meta-Analysis

**DOI:** 10.3390/jcdd12070263

**Published:** 2025-07-09

**Authors:** Dimitrios E. Magouliotis, Serge Sicouri, Massimo Baudo, Francesco Cabrucci, Yoshiyuki Yamashita, Basel Ramlawi

**Affiliations:** 1 Department of Cardiac Surgery Research, Lankenau Institute for Medical Research, Main Line Health, Wynnewood, PA 19096, USA; sicouris@mlhs.org (S.S.); massimo.baudo@icloud.com (M.B.); cabruccif@mlhs.org (F.C.); yamashitay@mlhs.org (Y.Y.); ramlawib@mlhs.org (B.R.); 2 Department of Cardiac Surgery, Lankenau Heart Institute, Main Line Health, Wynnewood, PA 19096, USA

**Keywords:** transcatheter aortic valve replacement, tavr, infective endocarditis, tavr explantation, surgical management

## Abstract

Background: Infective endocarditis after transcatheter aortic valve replacement (TAVR-IE) is a rare but severe complication associated with high morbidity and mortality. The optimal treatment strategy—surgical explantation versus medical therapy—remains uncertain, particularly given the technical demands of TAVR removal and the advanced age of many affected patients. Methods: We conducted a systematic review and meta-analysis of studies comparing the surgical and medical management of TAVR-IE. Primary outcomes included 30-day mortality and 1-year survival. Secondary analyses explored microbiological profiles, patient demographics, prosthesis type, postoperative complications, and surgical indications. A qualitative synthesis of surgical explantation techniques and reconstructive strategies was also performed based on recent consensus recommendations. Results: Three studies comprising 1557 patients with TAVR-IE were included; 155 (10.0%) underwent surgical treatment. Thirty-day mortality was comparable between groups (surgical: 9.7%; medical: 8.4%), while the pooled odds ratio for one-year survival did not reach statistical significance (OR: 1.91, 95% CI: 0.36–10.22; *I*^2^ = 88%). However, single-center outcomes demonstrated markedly improved survival with surgery (96% vs. 51%). The most common surgical indications included severe valvular dysfunction (50.3%), aortic root abscess (26.5%), and large vegetations (21.3%), in line with current guideline recommendations. Postoperative complications included acute renal failure (10%) and longer hospitalizations (19.8 vs. 18 days), although these were not statistically different. Contemporary explant strategies—such as the Double Kocher, Tourniquet, and Y-incision aortic enlargement techniques—were highlighted as critical tools for surgical success. Conclusions: While underutilized, surgical intervention for TAVR-IE may offer significant survival benefits in select patients, particularly when guided by established indications and performed at high-volume centers. Outcomes depend heavily on timing, surgical expertise, and appropriate patient selection. As TAVR expands to younger populations, TAVR-IE will become increasingly relevant, necessitating early multidisciplinary involvement and broader familiarity with advanced explant techniques among cardiac surgeons.

## 1. Introduction

Transcatheter aortic valve replacement (TAVR) has revolutionized the management of severe aortic stenosis, offering a minimally invasive alternative to surgical aortic valve replacement (SAVR), especially in patients with high or prohibitive surgical risk [1,2,3,4]. Over the past decade, its indications have expanded to include intermediate- and low-risk populations, leading to a dramatic rise in the number of procedures performed globally [5,6,7]. As TAVR becomes increasingly prevalent in younger and healthier patients, long-term complications such as prosthetic valve endocarditis—specifically, infective endocarditis after TAVR (TAVR-IE)—have emerged as a growing clinical concern [8]. Despite being relatively rare, with reported incidences ranging from 0.3% to 2.0% per 100 person-years, TAVR-IE is associated with dismal outcomes, including high in-hospital mortality rates of 30–40% and poor long-term survival [8]. Common causative organisms include *Staphylococcus Aureus* and *Enterococcus* species, often resulting in aggressive disease courses with embolic phenomena, persistent bacteremia, and prosthesis-associated abscess formation [8].

The management of TAVR-IE remains controversial and complex. While surgery is considered the cornerstone of therapy for prosthetic valve endocarditis in surgical valve recipients, its role in patients with TAVR-IE is less well-defined [8]. Several multicenter studies and registry analyses have reported low rates of surgical intervention—typically below 20%—despite a significant proportion of patients meeting guideline-based surgical indications [8]. This reluctance may stem from the high procedural risk of TAVR explantation, technical challenges due to neoendothelialization, and patient comorbidities [8]. However, emerging evidence suggests that selected patients with surgical indications may derive a significant survival benefit from timely surgical intervention.

Given the rapidly evolving landscape of TAVR and the increasing burden of TAVR-IE, there is a critical need to systematically evaluate the outcomes of surgical versus medical management in this high-risk population. This systematic review and meta-analysis aim to synthesize current evidence on the comparative effectiveness of these two treatment strategies for TAVR-IE, providing much-needed clarity to guide clinical decision-making in this challenging scenario.

## 2. Materials and Methods

### 2.1. Search Strategy and Article Selection

This meta-analysis was conducted following a predefined protocol that was approved by all authors. The study adhered to the Preferred Reporting Items for Systematic Reviews and Meta-Analyses (PRISMA) guidelines [9], ensuring methodological rigor. The protocol was registered in the Open Science Framework (OSF) Registries (registration doi: 10.17605/OSF.IO/W8MG7). The 2020 PRISMA Checklist is available in Table S1. To identify relevant studies, a comprehensive literature search was performed across PubMed (Medline), Scopus (ELSEVIER), and the Cochrane Central Register of Controlled Studies (CENTRAL), with the final search completed on 1 April 2025. The search strategy included various keywords such as “infective endocarditis”, “ie”, “transcatheter aortic valve replacement”, “tavr”, “tavi”, “explant”, “surgery”, “medical”, and “treatment”.

The inclusion criteria for this meta-analysis were as follows: (a) original studies with at least 10 patients, (b) published between 2000 and 2025, (c) written in English, (d) conducted on human subjects, and (e) reporting comparative outcomes for surgical and medical treatment for patients presenting with IE following TAVR. Duplicate publications were excluded. Additionally, the reference lists of all included studies were reviewed to identify further relevant articles. Two independent reviewers (DEM and SS) extracted data from the eligible studies. Any disagreements between the reviewers were resolved through discussion with the senior author (BR) until a consensus was reached. The authors maintained personal equipoise regarding the optimal surgical approach.

### 2.2. Data Extraction and Endpoints

For each selected study, data were collected on patient baseline characteristics, surgical details, and perioperative outcomes. The primary endpoints were (a) the in-hospital mortality median overall survival (OS) and the 1-year overall survival (OS). Secondary endpoints included the postoperative complications and the predictors of in-hospital mortality. A leave-one-out sensitivity analysis was carried out, in which the meta-analysis was repeated for each subset of studies, excluding one study at a time. This method assesses the influence of individual studies on the overall results.

### 2.3. Quality and Publication Bias Assessment

To assess the methodological quality of the non-randomized studies included in this meta-analysis, the Newcastle-Ottawa Quality Assessment Scale (NOS) was employed [10]. This tool assigns ratings on a scale from zero to nine stars, with studies receiving at least five stars considered to be of acceptable quality. Additionally, the Risk of Bias in Non-Randomized Studies of Interventions (ROBINS-I) tool was used to systematically evaluate potential biases within the included studies [11]. Since no randomized controlled trials (RCTs) were part of this analysis, all assessments were conducted independently by two reviewers (DEM and SS), with any discrepancies resolved through discussion and consensus.

### 2.4. Statistical Analysis

Meta-analytic calculations were performed using Review Manager (RevMan) version 5.4.1, developed by the Cochrane Collaboration (London, UK). Dichotomous outcomes, including 30-day mortality and 1-year survival, were analyzed using the Mantel-Haenszel method with a random-effects model to account for expected clinical and methodological heterogeneity across studies. Effect sizes were expressed as odds ratios (ORs) with 95% confidence intervals (CIs). Heterogeneity was assessed using the *I*^2^ statistic, with thresholds of 25%, 50%, and 75% representing low, moderate, and high heterogeneity, respectively. Statistical significance was defined as *p* < 0.05.

## 3. Results

### 3.1. Search Strategy and Patient Demographics

**Figure 1:**
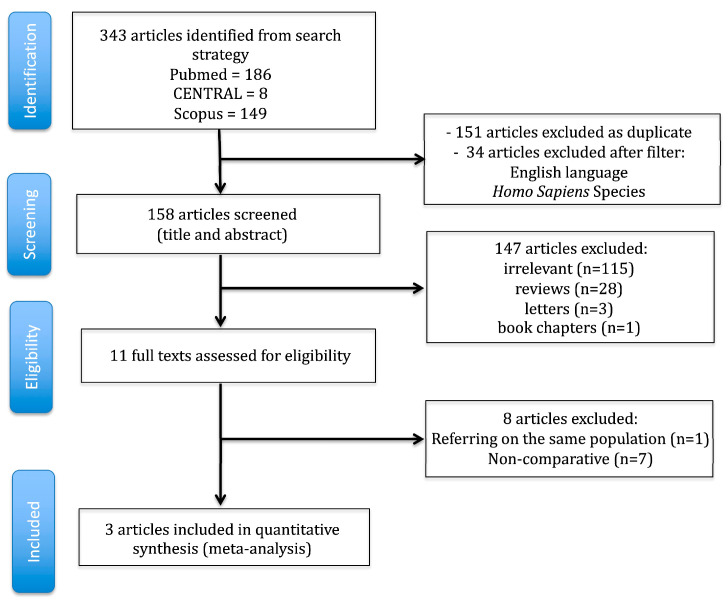
Literature search trial flow.

**Figure 2:**
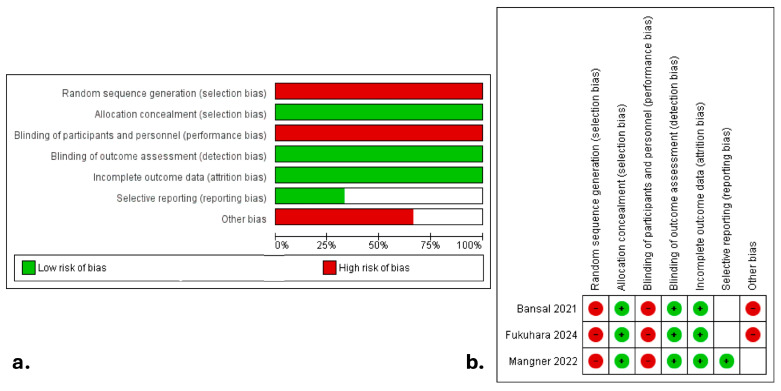
Risk of Bias in Non-Randomized Studies of Interventions with (**a**). summary plot and (**b**). traffic lights [12,13,14].

The literature search strategy is visually outlined in Figure 1, while the characteristics of the included studies are detailed in Table 1. A total of 343 articles were retrieved from PubMed, Scopus, and Cochrane Central Register of Controlled Trials (CENTRAL), with three studies [12,13,14] ultimately meeting the inclusion criteria for both qualitative and quantitative synthesis. The inter-reviewer agreement was deemed “almost perfect” (kappa = 0.91; 95% CI: 0.81–1.00). The ROBINS-I tool assessment, presented in Figure 2a,b, highlighted selection and performance biases as the primary methodological concerns. All the studies were retrospective [12,13,14]. No randomized controlled trials (RCTs) were available for inclusion in this meta-analysis, due to the nature of the investigated topic. Two studies were conducted in the USA [12,13], and one was multinational [14], spanning from 2021 to 2024.

A total of three studies met the inclusion criteria, encompassing 1557 patients diagnosed with infective endocarditis (IE) after transcatheter aortic valve replacement (TAVR). Of these, 155 patients (10%) were managed surgically, while 1402 (90%) received medical therapy alone. Balloon-expandable prostheses were used in 57% of surgical patients versus 50% of medical patients (*p* = 0.17). Surgically treated patients were younger (median age 74 vs. 79 years) and less frequently female (23% vs. 40%). Chronic kidney disease and pacemaker rates were similar across groups. The most common pathogens were *Enterococcus* species (25%), *Staphylococcus Aureus* (22%), and *Streptococcus* species (21%). The prevalence of *S. Aureus* and *Enterococcus* was comparable between groups, while *Streptococcus* infections were significantly more frequent in the medical cohort (*p* < 0.001). Surgical intervention was predominantly pursued in accordance with guideline-based criteria. The most frequent indications included severe valvular dysfunction (50%), aortic root abscess (27%), and large vegetations (21%), followed by persistent bacteremia (10%), multivalve involvement (10%), and conduction abnormalities (1.3%). Approximately 44% of the surgically treated patients underwent a concomitant or combined procedure. The most commonly performed concomitant interventions included aortic root replacement, mitral valve repair or replacement, ascending aortic repair, and coronary artery bypass grafting (CABG). Additionally, tricuspid valve interventions were reported in selected cases. Table 1 presents baseline patient characteristics, while Table 2 outlines the primary pathogen distribution.

### 3.2. Primary Endpoint: 30-Day Mortality and 1-Year Survival

**Figure 3:**
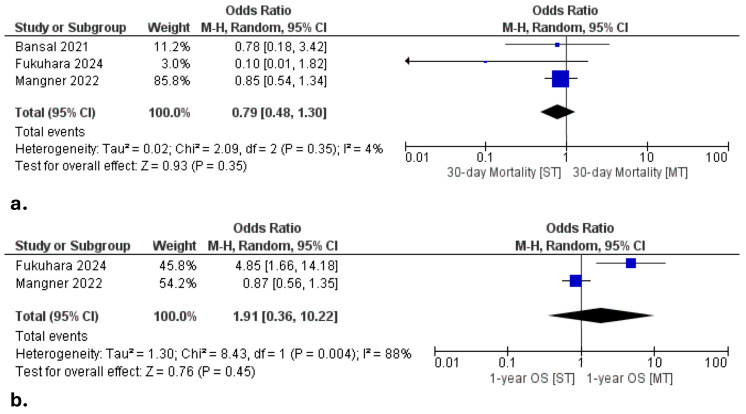
Forest plots comparing the surgical treatment (ST) versus medical treatment (MT) group in terms of (**a**). 30-day mortality and (**b**). 1-year survival [12,13,14].

Among surgically treated patients, the pooled 30-day mortality was 9.7% (15/155; 95% CI: 6.0–15.4%). In comparison, the 30-day mortality in the medically managed cohort was 8.4% (118/1402; 95% CI: 7.1–10.0%). Mortality rates were comparable between groups, although wide confidence intervals and significant inter-study heterogeneity precluded definitive conclusions (Figure 3a).

The pooled odds ratio for 1-year survival did not reach statistical significance (OR 1.91, 95% CI: 0.36–10.22; *p* = 0.45), with substantial inter-study heterogeneity (*I*^2^ = 88%). In the Fukuhara et al. cohort, surgical patients exhibited significantly higher survival at 1 year compared to those medically managed (96% vs. 51%, OR 4.85, 95% CI: 1.66–14.18). However, no difference was observed in the Mangner et al. [14] cohort (OR 0.87, 95% CI: 0.56–1.35), which contributed to the overall non-significant pooled effect. These findings suggest potential benefit from surgery in select patients, but underscore the variability in outcomes across study populations.

### 3.3. Postoperative Complications and Predictors of In-Hospital Mortality

Postoperative complications among surgically treated patients included acute kidney injury in 40.0%, stroke in 9.6%, and dialysis requirement in 1.5%. Septic shock occurred in 27.9% of cases, systemic embolism in 18.9%, and major bleeding in 5.4% (Table 3). Blood transfusions were reported in 6.6% of surgical cases, while persistent bacteremia was noted in 44.1%. Notably, all patients who required temporary renal replacement therapy recovered baseline renal function. Length of hospitalization was slightly longer in surgical patients compared to those managed medically (19.8 vs. 18.0 days), though this difference was not statistically significant (*p* = 0.525).

Predictors of in-hospital mortality varied across studies (Table 4). In the Bansal cohort, dialysis during hospitalization, acute kidney injury, cardiogenic shock, *Staphylococcus Aureus* infection, stroke, and female sex were all associated with significantly increased risk. The Mangner study [14] identified septic shock (OR: 3.09), systemic embolism (OR: 2.41), *S. Aureus* infection (OR: 2.03), and increasing age (OR: 1.04 per year) as significant predictors. Importantly, surgical management itself was associated with a reduced risk of in-hospital mortality in their cohort (OR: 0.51; 95% CI: 0.27–0.96), suggesting a protective effect of early operative intervention in appropriate candidates.

### 3.4. Quality and Publication Bias Assessment

The quality assessment of the included studies using the Newcastle-Ottawa Scale (NOS) is summarized in Table 1. While all three studies were observational in design, they demonstrated moderate to high methodological quality based on cohort selection, comparability, and outcome assessment. Figure 2 illustrates the risk of bias assessment using the ROBINS-I tool. The primary areas of concern were related to confounding, particularly selection bias inherent in retrospective designs and variation in the criteria for surgical referral. Nevertheless, outcome data were clearly defined and consistently reported across studies.

Heterogeneity for the primary endpoints—30-day mortality and 1-year survival—was variable. While 30-day mortality exhibited low heterogeneity, 1-year survival was associated with substantial heterogeneity (*I*^2^ = 88%), likely reflecting differences in institutional expertise, timing of intervention, and thresholds for surgical eligibility. Secondary endpoints such as length of stay and postoperative renal failure also showed clinical variability, driven by differences in perioperative care protocols, valve types, and patient profiles across institutions.

Visual inspection of the funnel plot revealed asymmetry, suggesting the presence of publication bias, particularly due to potential underreporting of small studies with neutral or unfavorable surgical outcomes. However, given the limited number of available studies (*n* = 3), this asymmetry may also be attributed to small-sample artifacts rather than true reporting bias. Importantly, the robustness of our results is reinforced by the use of a random-effects model and the clinical consistency observed in surgical indications and postoperative trends across the cohorts.

## 4. Discussion

This systematic review consolidates the available literature comparing surgical and medical management in patients with infective endocarditis following TAVR. Although rare, TAVR-IE carries a devastating prognosis and poses unique diagnostic and therapeutic challenges [8,15,16]. Despite frequent surgical indications, operative intervention remains uncommon in clinical practice. Our findings reveal a consistent pattern of underutilization of surgery, even in patients who would typically meet criteria for intervention in the setting of prosthetic valve endocarditis.

The underutilization of surgery in TAVR-IE is multifactorial. First, these patients are often initially managed by interventional cardiologists, and referral to cardiac surgeons may occur only after significant clinical deterioration, thus effectively positioning the surgical team as a “second-line” consultant. Secondly, the explantation of transcatheter valves poses certain technical challenges that go beyond those of conventional reoperations on surgical prostheses. The degree of neoendothelialization, annular involvement, and patient frailty frequently render these cases more complex than standard redo operations for prosthetic valve endocarditis, thereby elevating perceived and actual operative risk [17].

While 30-day mortality was comparable between surgical and medical groups (~9%), the pooled analysis of 1-year survival did not reach statistical significance (OR 1.91, 95% CI: 0.36–10.22; *p* = 0.45), with substantial inter-study heterogeneity (*I*^2^ = 88%). However, the single-center study by Fukuhara et al. [13] reported a striking survival advantage in favor of surgery (96% vs. 51%, OR: 4.85, 95% CI: 1.66–14.18), likely reflecting earlier intervention and management at a high-volume center. In contrast, the larger multicenter registry by Mangner et al. [14] demonstrated no significant difference (OR 0.87, 95% CI: 0.56–1.35), contributing to the overall neutral pooled result. These contrasting outcomes underscore the importance of institutional experience, patient selection, and surgical timing. Rather than dismissing the potential benefit of surgery, the variability observed highlights a more nuanced reality: surgical benefit in TAVR-IE is likely context-specific, dependent on individualized decision-making, early multidisciplinary engagement, and referral to experienced centers. Another critical observation is the stark variability in the proportion of patients undergoing surgery across the included studies—ranging from 2.5% in the Mangner registry to over 50% in the Fukuhara single-center experience. This discordance likely reflects institutional philosophy, surgical availability, referral timing, and perhaps selection bias. It also underscores the absence of uniform criteria for surgical referral and candidacy in TAVR-IE, a gap that highlights the urgent need for consensus-based triage pathways and structured Endocarditis Team involvement.

Surgical explantation of transcatheter valves is undoubtedly complex. Neoendothelialization, annular invasion, and device integration into native structures might challenge even the most experienced surgical teams. National registry data have demonstrated frequent requirements for aortic root or multivalve procedures, with 30-day mortality reaching nearly 20% in patients undergoing concomitant interventions [18]. However, in experienced centers, such as that reported by Fukuhara et al. [13], mortality can be driven to 0% with excellent intermediate-term outcomes, reinforcing the importance of institutional experience and case selection. Patients undergoing surgery were generally younger and less often female. Although these trends likely reflect cautious surgical selection, they may also introduce confounding when comparing outcomes. Importantly, device type (balloon vs. self-expandable) did not significantly impact surgical mortality, challenging any presumption that certain prosthesis types are inherently “unsuitable” for surgical rescue.

Predictors of in-hospital mortality provide crucial insight into the risks associated with surgical management of TAVR-IE and underscore the importance of early intervention. In the Bansal et al. study [12], key independent predictors included dialysis during hospitalization, acute kidney injury, cardiogenic shock, and *Staphylococcus aureus* infection, with stroke and female sex also contributing to worse outcomes. These factors reflect both the severity of infection and the physiological toll of delayed or inadequate treatment. Notably, several are potentially modifiable with timely surgical referral and aggressive preoperative optimization. Furthermore, a particularly intriguing finding in the surgical cohort was the high incidence of persistent bacteremia, reported in 44% of patients. While this might initially raise concerns about treatment failure, it may instead reflect the high disease burden, delayed intervention, or residual infectious foci in complex cases. Interestingly, some reports have described the persistence of bacteremia postoperatively not merely as a complication but as a potential therapeutic signal, thus guiding prolonged antibiotic regimens or identifying the need for reintervention [19]. In our pooled cohort, renal failure was the most common complication, yet dialysis was infrequently required, and full renal recovery was observed in high-volume centers. Recognizing these predictors can inform clinical triage and reinforce the role of multidisciplinary teams in selecting candidates for surgery before irreversible decompensation occurs.

As TAVR explantation becomes more frequent, especially in the context of endocarditis, familiarity with specific explant techniques is crucial for successful outcomes. The recent Heart Valve Collaboratory consensus has established technical guidelines for safe and effective TAVR removal based on prosthesis type, anatomy, and degree of integration into native tissues [20]. In this context, explantation of balloon-expandable valves (BEVs)—such as the Edwards Sapien series—can be performed using the Double Kocher technique or Roll technique, which rely on radial inward collapse of the stent frame after partial circumferential dissection [20,21]. These methods allow safe mobilization while minimizing trauma to the aorta or annulus. For self-expanding valves —such as the Medtronic Evolut series—the Tourniquet technique uses silk snaring of the frame through a pump tubing to reduce valve profile and facilitate atraumatic removal [20,21]. Additional strategies like the Handlebar Mustache technique (radial infolding after transverse division) offer alternative solutions for difficult SEV explants (Table 5).

For both BEVs and SEVs, preoperative multidetector computed tomography (CT) is essential for assessing prosthesis position, coronary anatomy, and potential adhesions or device integration with critical structures [22,23]. The complexity of explantation often scales with valve dwell time and endothelization; thus, early explants (e.g., for endocarditis) are typically easier than late explants for structural degeneration. In some cases, aortic root replacement or enlargement is required—particularly when annular involvement, periannular abscess, or prosthesis–patient mismatch is present [24]. Finally, when concomitant valve surgery is needed, such as mitral valve replacement, surgeons must be prepared for complex reconstructions involving the aortomitral curtain. Innovative hybrid techniques like SURPLUS (Surgical resection of prosthetic valve leaflets under direct vision)—where the stent frame is left intact while the leaflets are resected and replaced with a balloon-expandable valve—are emerging as alternatives for selected high-risk patients, though they are contraindicated in endocarditis [21].

In cases where TAVR prostheses are explanted in patients with a small annulus or prior mismatch, advanced annular enlargement techniques may be required to facilitate proper prosthetic valve sizing. The Y-incision aortic annular enlargement (AAE) technique offers a robust strategy for upsizing the annulus following transcatheter valve explantation [24]. In the context of TAVR explant, this method involves extending the incision into the aortomitral curtain and placing a tailored Hemashield patch in an “Arc and Roof” configuration to increase annular diameter and support root reconstruction [24]. The procedure enabled implantation of a significantly larger surgical valve (from annulus 19 mm to size 25), with excellent hemodynamic outcomes and no postoperative paravalvular leak [24]. Y-incision AAE has been associated with a median increase of three valve sizes, a valve area of 2.3 cm^2^, and minimal residual gradients, making it a valuable technique for surgeons facing complex explant scenarios [24]. Given these advantages, this approach may be considered a preferred reconstructive option in cases of annular destruction, prior undersizing, or anticipated need for future valve-in-valve intervention.

The diversity of techniques reflects both the heterogeneity of transcatheter valve designs and the growing body of surgical expertise in managing their failures. As TAVR expands into younger populations, technical mastery of explant strategies will be essential for modern valve surgeons. While medical therapy remains appropriate in patients without surgical indications or those deemed inoperable after multidisciplinary review, a shift in surgical referral patterns may be warranted. Cardiac surgeons must advocate for early involvement in TAVR-IE cases—particularly when guideline-based indications are present—before irreversible decompensation limits therapeutic options.

Beyond the technical aspects of surgical explantation, the involvement of dedicated Endocarditis Teams has emerged as a critical factor in optimizing outcomes. These multidisciplinary groups, comprising cardiologists, cardiac surgeons, infectious disease specialists, microbiologists, and imaging experts, can streamline decision-making, ensure adherence to guideline-based criteria, and facilitate timely intervention. Notably, El-Dalati et al. found mortality dropped from 29.4% to 7.1% after their implementation [25], while a 2023 meta-analysis also confirmed lower short-term mortality with such teams [26]. Prospective data show diagnostic or therapeutic plans were modified in up to 42% of patients reviewed by these teams [26]. Moreover, long-term mortality was halved following team-based protocols in earlier work by Botelho-Nevers et al. [27]. The role of Endocarditis Teams is particularly vital in navigating the complexity of TAVR-IE, where individualized treatment plans are paramount. As such, broader implementation of Endocarditis Teams may improve surgical referral patterns and ultimately enhance patient survival. As TAVR becomes increasingly common, particularly among lower-risk and younger patients, we are witnessing the emergence of a new and complex patient population: those with prosthetic endocarditis in whom standard surgical rescue is often deemed too risky. This phenomenon raises both clinical and ethical concerns, as guideline-based treatment is frequently withheld despite potential benefit. The findings of this review not only inform clinical outcomes but also call for systemic changes in referral patterns, multidisciplinary evaluation, and earlier surgical consultation to ensure that high-risk does not become synonymous with therapeutic nihilism.

This review is limited by the small number of included studies (*n* = 3), all of which were observational and retrospective. Patient-level data were not available, precluding time-to-event analysis or adjustment for baseline characteristics. The pooling of results was limited by inter-study heterogeneity in endpoints, reporting, and sample size. Additionally, length of stay and complication rates were approximated based on summary statistics. While trends were notable, statistical power was insufficient to detect significance across all comparisons. Lastly, publication bias may have led to underreporting of negative or non-surgical series.

## 5. Conclusions

Infective endocarditis following transcatheter aortic valve replacement (TAVR-IE) is a rare but devastating complication that demands nuanced decision-making and technical expertise. Despite the presence of guideline-based indications in many patients, surgery remains underutilized, often due to concerns over operative risk or delayed referral. This review suggests that when performed early and in experienced centers, surgical explantation may offer meaningful survival benefits without prohibitive perioperative risk. While the meta-analysis did not show a statistically significant improvement in 1-year survival, single-center outcomes highlight the critical role of timely intervention and tailored surgical strategy. As the field advances, techniques such as root replacement and Y-incision annular enlargement will be increasingly relevant. Ultimately, managing TAVR-IE requires a shift in mindset—away from futility and toward early, aggressive, and individualized care.

## Figures and Tables

**Table 1:** Baseline characteristics of studies finally included in the meta-analysis.

Study ID, Year	Country	Study Design	Patients, *n*	Age, Years	Female Sex, %	DM, %	Dialysis, %	Previous CVA, % ST/MT	Previous CS, %	Pacemaker, % ST/MT	Balloon-Type Prosthesis, % ST/MT	NOS
			ST/MT	ST/MT	ST/MT	ST/MT	ST/MT		ST/MT			
Bansal 2022 [12]	USA	R	20/886	72 (70–79)/81 (73–87)	26/40	36/35	0/8	0/11	N/A	N/A	N/A	6
Fukuhara 2024 [13]	USA	R	24/43	71 (60–81)/76 (68–83)	13/40	63/44	21/19	25/12	38/23	42/33	58/33	6
Mangner 2022 [14]	Multinational	R-IP	111/473	78 (74–82)/81 (76–86)	31/39	32/38	35/45	17/12	22/23	4/2	57/52	7

The Newcastle-Ottawa Scale (NOS) for assessing the quality of non-randomized studies. The highest quality studies are awarded up to 9 stars. Abbreviations: ST = Surgical Treatment; MT = Medical Treatment; N/A = Not Available.

**Table 2:** The distribution of pathogens across studies.

Study ID, Year	*S. aureus*, % ST/MT	*Enterococcus*, % ST/MT	*Streptococcus*, % ST/MT	CoNS, % ST/MT
Bansal 2022 [12]	50/21	0/25	16/25	3/0
Fukuhara 2024 [13]	13/12	5/10	6/5	5/7
Mangner 2022 [14]	2/5	N/A	1/1	4/5

Abbreviations: *S. aureus* = *Staphylococcus aureus*; CoNS = Coagulase Negative *Staphylococci*; ST = Surgical Treatment; MT = Medical Treatment.

**Table 3:** Postoperative Complications in the Surgical Cohort.

Complication	Frequency, %
Acute Kidney Injury	40
Dialysis	2
Stroke	10
Renal Recovery	100
Cardiogenic Shock	9
Blood Transfusion	7
Major Bleeding	5
Systemic Embolism	19
Septic Shock	28
Persistent Bacteremia	44
In-hospital Mortality	24

**Table 4:** Major predictors of in-hospital mortality.

Predictor	Bansal [12] OR (95% CI)	Mangner [14] OR (95% CI)
Dialysis During Hospitalization	6 (2–15)	-
Acute Kidney Injury	5 (3–10)	-
Cardiogenic Shock	5 (1–21)	-
*S. Aureus* Infection	3 (2–6)	2 (1–4)
Stroke	3 (1–10)	Not Significant
Female	2 (2–4)	-
Septic Shock	-	3 (2–6)
Systemic Embolism	-	2 (1–5)
Age	-	1 (1–1)
Surgical Management (protective)	-	1 (0–1)

No available data regarding the Fukuhara et al. study [13].

**Table 5:** Sum of the TAVR explantation techniques.

Valve Type	Technique	Description
Balloon-expandable (BEV)	Double Kocher	Circumferential dissection with radial inward collapse using two Kocher clamps.
	Roll Technique	Gradual rolling of the stent inward after leaflet and frame mobilization.
Self-expanding (SEV)	Tourniquet Technique	Silk suture loop through valve frame, tightened with pump tubing to collapse device.
	Handlebar Mustache	Transverse frame division with radial infolding for extraction.
Both types	CT Planning	Multidetector CT to evaluate device position, integration, and coronary anatomy.
	Root Replacement	Required in cases of annular destruction, abscess, or significant PVL.
Hybrid/Advanced	SURPLUS (Leaflet Resection)	Resection of infected leaflets with stent frame left in place (not for IE).

## Data Availability

Supporting raw data is available upon request.

## Supplementary Materials

The following supporting information can be downloaded at https://www.mdpi.com/article/10.3390/jcdd12070263/s1: Table S1: PRISMA checklist.
